# BRIDGING THE GAP IN OBESITY TREATMENT FOR OLDER PEOPLE: THE ROLE OF THE TREAT AND REDUCE OBESITY ACT (TROA)

**DOI:** 10.1093/geroni/igae098.3933

**Published:** 2024-12-31

**Authors:** Samuel C Van Vleet, Jeein Jang, Maizonne Fields

**Affiliations:** Miami University, Oxford, Ohio, United States; University of Massachusetts, Boston, Boston, Massachusetts, United States; The University of Alabama at Birmingham, Birmingham, Alabama, United States

## Abstract

Obesity is a chronic disease with significant impacts on health outcomes and economic burdens throughout the life course. Obesity is prevalent and often undertreated among older people, especially those with lower socioeconomic status and those living in rural areas. Current coverage for obesity treatment is sparse and inconsistent across states, creating barriers to care for many. The Treat and Reduce Obesity Act (TROA), a public policy initiative that has been in development for over a decade, seeks to address these gaps in care among Medicare beneficiaries. TROA, which has garnered bipartisan support and is bicameral, aims to improve access to comprehensive treatment options for older people. This presentation explores the benefits of treating obesity on various comorbidities, such as heart disease, sleep apnea, and diabetes, and will emphasize the need for expanded Medicare coverage for obesity treatments. Utilizing data from the 2024 Obesity Coverage Nexus Platform, we highlight the high prevalence and associated risks of obesity among older people and examine how state-level variations in insurance coverage for obesity treatments impact access. The passage of TROA would provide a more equitable and effective approach to obesity management, helping to alleviate the economic burden of out-of-pocket costs for obesity treatments and improving health outcomes for lower-income and underserved populations.

